# A study on cortical habituation based on event-related potential P50 and CNV

**DOI:** 10.3389/fneur.2026.1787063

**Published:** 2026-04-14

**Authors:** Jing Li, Yongxiang Zhang, Huanyu Li, Chang Liu, Zhiyuan Sun, Yunbo Fu, Qinghua He, Yudan Lv

**Affiliations:** 1 Neuroscience Center, Department of Neurology, The First Hospital of Jilin University, Changchun, China; 2 Headache Center, Department of Neurology, Beijing Tiantan Hospital, Capital Medical University, Beijing, China; 3 Changchun Institute of Optics, Fine Mechanics and Physics, Chinese Academy of Sciences, Changchun, China; 4 State Key Laboratory of CNS/ATM & MIIT Key Laboratory of Complex-field Intelligent Sensing, Beijing Institute of Technology, Beijing, China; 5 Guangdong Province Key Laboratory of Intelligent Detection in Complex Environment of Aerospace, Land and Sea, Beijing Institute of Technology, Zhuhai, China

**Keywords:** CNV, event-related potential, fast fourier transform, migraine, P50

## Abstract

This study systematically evaluated electrophysiological alterations in early sensory gating and late cognitive anticipation functions in migraine patients using the P50 and Correlated Negative Variation (CNV) components of event-related potentials (ERPs). Findings revealed: - Under the P50 paradigm, patients exhibited prolonged latencies and increased suppression rates, indicating impaired sensory gating. - Under the CNV paradigm, patients demonstrated prolonged latencies across multiple components, along with significantly increased occipital iCNV amplitude and CNV area values, reflecting slowed information processing and heightened cortical excitability. - Enhanced theta and alpha band energy was observed across extensive brain regions in both paradigms. These results indicate that migraine patients exhibit impaired cortical inhibitory function and abnormal information processing throughout the entire pathway, from early sensory gating to late cognitive anticipation. This electrophysiological profile provides an objective, quantitative neurobiological marker for migraine-related cognitive impairment, holding potential value for clinical diagnosis and monitoring.

## 1 Introduction

Migraine is a common chronic neurological disorder and the second leading cause of neurological disability worldwide (1). It affects approximately 1.1 billion people and significantly impairs their quality of life (2). It is characterized by recurrent unilateral or bilateral throbbing headaches (3). Its pathophysiological mechanisms involve cortical spreading depression (CSD), activation of the trigeminal vascular system, and central sensitization (3). Research indicates the underlying cause is an imbalance of excitatory and inhibitory neural activity within the brain (4, 5).

Beyond headache, migraine sufferers frequently experience cognitive impairments such as inattention and slowed response times during or between attacks (6–8). However, commonly used cognitive screening tools like the Montreal Cognitive Assessment (MoCA) and Mini-Mental State Examination (MMSE) are limited by low sensitivity and high false-negative rates in the migraine crowd (9, 10). Consequently, researchers have increasingly turned to alternative assessment methods, particularly in the migraine without aura subtype, aiming to more accurately elucidate the relationship between migraine and cognitive impairment (11). Event-related potentials (ERP) objectively reflect information processing within the cerebral cortex (12). Within ERP research, the auditory sensory gating potential P50 assesses early sensory gating function, reflecting the brain’s automatic filtering capacity for irrelevant stimuli (13, 14); Concurrently, the contingent negative variation (CNV) correlates with temporal anticipation, sustained attention, and action readiness, reflecting higher-order cognitive functions in prefrontal cortex regulation during anticipatory processes (15, 16). P50 and CNV, respectively, provide complementary electrophysiological evidence for migraine-related brain abnormalities across two distinct temporal phases: early sensory filtering and late cognitive anticipation (17, 18). Furthermore, quantitative analysis of P50 and CNV data via Fast Fourier Transform (FFT) generates EEG power spectral density plots, which enable comparison of topographical variations in brain activity (19, 20). Research indicates that migraine sufferers frequently exhibit cortical hyperreactivity and impairs adaptation to repetitive stimuli (21). As a fundamental form of implicit learning, habituation enables organisms to disregard persistent irrelevant or non-threatening stimuli—a crucial mechanism that allows the brain to adapt effectively to its environment (22, 23). P50 serves as an indicator for assessing early sensory gating function, evoked by dual-tone stimulation (S1 and S2), reflecting the central nervous system’s ability to inhibit repetitive auditory stimuli (24). Previous studies have indicated that P50 gating exhibits increased amplitude in patients with mild cognitive impairment (MCI) (25). Since migraine patients often suffer from cognitive decline, P50 can be used to study early sensory gating function in migraine patients. This paradigm typically presents two identical auditory stimuli (70–80 dB) at a 500-millisecond interval, requiring participants to attend to the stimuli (26, 27). In healthy individuals, the P50 amplitude evoked by S2 is typically lower than that of S1, with the S2/S1 ratio often below 0.5 (28). This indicates the brain’s ability to suppress redundant information and maintain normal sensory gating effectively. In contrast, migraine patients exhibit insufficient reduction in S2 amplitude and an elevated S2/S1 ratio, suggesting impaired sensory gating mechanisms. Further studies reveal that P50 testing can distinguish sensory gating differences between migraine patients and healthy controls (29). For instance, M. Siniatchkin’s research indicates a significantly increased S2/S1 amplitude ratio in migraine patients, suggesting abnormal short-term adaptation (28). Song’s research indicates that patients with mild cognitive impairment also exhibit increased S2/S1 amplitude ratios (25). Given that migraine patients often present with clinical cognitive decline, these findings suggest migraine and cognitive impairment may share similar neurophysiological underpinnings in sensory processing mechanisms. Furthermore, the occasional negative variation (CNV)—an ERP assessing cognitive anticipation—exhibits abnormally amplified negative amplitudes in migraine patients, encompassing initial CNV (iCNV), terminal CNV (tCNV), and overall CNV (oCNV) (16). Even during pain-free intervals, patients maintain elevated oCNV negative amplitudes, indicating persistent cortical excitability abnormalities and deficits in cognitive anticipation function (30). For example, Kropp et al. found that patients with migraine without aura exhibited higher negative oCNV amplitude scores between attacks compared to healthy controls, suggesting that central adaptive dysfunction may persist even during asymptomatic phases.

Research indicates that migraine sufferers frequently exhibit abnormal P50 and CNV patterns: diminished P50 habituation suggests impaired sensory gating, whilst amplified CNV amplitude reflects heightened cortical excitability and disrupted cognitive modulation (25, 31). However, studies integrating both P50 and CNV remain scarce, and the underlying mechanism of inhibitory-excitatory imbalance remains unclear (32–34). To address this, the present study employs P50 and CNV experimental paradigms to systematically evaluate cortical functional states in migraine patients from sensory gating to cognitive anticipation stages. FFT analysis of EEG power spectrum characteristics will detect abnormal activity in local brain regions. This research aims to elucidate the underlying mechanism of depression and habituation deficits in migraine patients at the neurophysiological level, providing novel theoretical insights into their cognitive impairments.

## 2 Methods

This study was approved by the Ethics Committee of the First Hospital of Jilin University, with all participants providing signed informed consent.

### 2.1 Study population

Individuals meeting the inclusion criteria were selected from patients diagnosed with migraine who attended Jilin University First Hospital between October 2024 and June 2025, forming the Migraine group. Thirty age-, gender-, and Education-matched healthy volunteers were recruited to form the HC group. Patients were required to maintain a headache diary (recording duration, severity, and monthly frequency of attacks, with severity assessed via VAS) and complete a structured demographic questionnaire covering headache characteristics, medical history, and medication use. All participants underwent assessment using the MSE, MOCA, Hamilton Anxiety Scale (HAMA), and Hamilton Depression Scale (HAMD). Any individuals deemed questionable were subject to further reassessment by a psychiatrist.

#### 2.1.1 Inclusion criteria

1. Participants met diagnostic criteria for migraine without aura (ICHD-3);
2. Participants with migraine without aura had no concomitant headache disorders such as cluster headache, tension-type headache, or other psychiatric conditions;
3. Participants aged between 30 and 70 years with an educational attainment of primary school level or above;
4. HAMA score ≤ 7; HAMD score ≤ 8;
5. No use of psychotropic medications within the preceding two weeks;
6. All participants were right-handed with no history of smoking, alcohol consumption, or substance abuse.

#### 2.1.2 Exclusion criteria

1. Headache caused by organic disease, severe anxiety, or depression;
2. Participants with neurological impairment, such as cerebral hemorrhage, traumatic brain injury, brain tumor, or prior brain surgery;
3. Cognitive impairment confirmed by MMSE or MOCA;
4. Severe hearing impairment;
5. Subjects taking psychotropic medications or consuming foods that may trigger headache attacks during the study period, such as sedatives, antiepileptic drugs, coffee, or tea;
6. Participants receiving prophylactic anti-migraine treatment;
7. Participants were unable to cooperate sufficiently to complete the study.

### 2.2 Event-related potentials and resting-state EEG acquistion

#### 2.2.1 ERP recording

Prior to the P50 and CNV recordings and Visual Analog Scale (VAS) assessment, participants completed the MMSE, MoCA, HAMA, and HAMD tests to exclude cognitive impairment, anxiety, and depression (Table 1). The experiment was conducted in the brain function examination room, employing a Neuroscan 64-channel EEG system for data acquisition. On the day preceding the experiment, participants were instructed to ensure adequate rest and clean their scalps. During the experiment, subjects sat in comfortable chairs wearing a 64-channel electrode cap. Electrodes were positioned according to the international 10–20 system, with the ground electrode placed at the tip of the nose. Eye-tracking electrodes were positioned to record horizontal and vertical eye movements. Electrode impedance was maintained below 10 kΩ, with a sampling rate of 1,000 Hz. Before recording, subjects were instructed to remain relaxed, focused, and minimize head movement. They sequentially performed P50 and CNV auditory tasks. Data underwent preprocessing using Curry 7 software, employing whole-brain averages as a reference to remove eye movement artifacts, interference artifacts, and 50 Hz power line interference. Processed data were archived for subsequent analysis.

**Table 1:** Evaluation of the general data between migraine and control groups.

General clinical data	Migraine	Control
Sample quantity	30	30
Age (years)	39.50 ± 16.51	38.88 ± 14.08
Male (%)	30.00	33.33
Female (%)	70.00	66.67
HAMA	4.86 ± 1.29	4.62 ± 1.40
HAMD	4.21 ± 1.25	4.81 ± 1.10
Pain intensity (VAS)	8.19 ± 0.78	-
Frequency of headache (per month)	9.19 ± 1.78	-
Education	13.02 ± 3.99	13.71 ± 3.40
Drug use during the study	-	-

HAMA, Hamilton Anxiety Rating Scale; HAMD, Hamilton Depression Rating Scale; VAS, Visual Analog Scale.

#### 2.2.2 P50 paradigm

The P50 auditory gating paradigm employed a classical paired-click stimulus-test design. Stimuli were presented binaurally via in-ear headphones as two identical clicks (S1 and S2), each with an intensity of 80 dB SPL and duration of 0.04 ms. The interstimulus interval between S1 and S2 was 500 ms, with a 10-s interval between stimulus pairs. The experiment comprised 60 valid stimulus pairs. Throughout the task, participants were instructed to remain awake, keep their eyes open, sit still, and passively listen to the auditory stimuli while continuous EEG data were recorded.

#### 2.2.3 CNV paradigm

CNV was measured across 40 trials. Each trial consisted of an alerting stimulus (S1; 50 Hz, 1,000 ms) and a command stimulus (S2; 50 Hz, maximum 3,000 ms), with an interstimulus interval of 3,000 ms between them. Participants were required to press a key immediately upon S2 presentation. Recording commenced 200 ms before S1 and concluded 4,500 ms after S2. 2.3 Data Analysis Methods.

#### 2.2.4 ERP data analysis

##### 2.2.4.1 Preprocessing

All ERP data were analyzed using Curry 7 software. Continuous EEG data were first processed with a 0.5–45 Hz bandpass filter to eliminate high- and low-frequency noise. Ocular artifacts, muscle activity, and 50 Hz power line interference were removed using whole-brain average referencing. Trials with amplitudes exceeding ±100 μV were excluded from further analysis. For both paradigms, individual averages were computed for each condition, and all measurements were derived from these averaged waveforms.

##### 2.2.4.2 P50 analysis

For the P50 task, data were epoched into 1,500-ms segments spanning from 500 ms pre-S1 to 500 ms post-S2 (600 data points per epoch at 2.5-ms resolution), with baseline correction applied using the 500-ms pre-S1 interval. Individual averages were computed separately for S1 and S2 responses. S1 P50: Defined as the most positive peak occurring between 30 and 90 ms post-S1. Latency was measured at the peak of this component, with reference to the subsequent N1 peak for verification. S2 P50: Identified within a 10-ms window centered on the individual’s S1 P50 latency. Amplitude: Peak amplitudes for both S1 and S2 P50 were measured relative to the pre-stimulus baseline. P50 suppression ratio (S2/S1) was calculated for each participant as a measure of sensory gating. These ratios were then compared across groups to assess differences in sensory gating function.

##### 2.2.4.3 CNV analysis

For the CNV task, continuous EEG data were segmented into epochs from 200 ms pre-S1 to 4,500 ms post-S2, using the 200-ms pre-S1 interval as baseline. Only trials with correct behavioral responses (key press after S2) were included in the averages. The following parameters were derived from the averaged waveforms: Amplitude: Mean amplitude of the oCNV (the entire S1–S2 interval), iCNV (mean amplitude within a ± 100 ms window centered on the peak negativity between 550–750 ms post-S1), and tCNV (mean amplitude over the 200 ms preceding S2); Latency: Latency of Point A (the positive-to-negative baseline crossing following S1), Point C (the negative-to-positive baseline crossing following S2), and the iCNV (the time point of the peak amplitude within the 550–750 ms post-S1 window); CNV Square: This metric was calculated for oCNV, iCNV, tCNV and PINV via the trapezoidal rule CNV Square = *Σ*[(Vᵢ + Vᵢ₊₁) × Δt/2], where Vᵢ and Vᵢ₊₁ represent baseline-corrected EEG amplitudes (μV), Δt is the sampling interval (ms), and the unit is μV·ms; oCNV, iCNV and tCNV CNV Square values were computed over the same time windows as their corresponding amplitude measures, PINV CNV Square was defined as the integrated negative potential between S2 onset and Point C, and all CNV Square measures were baseline-corrected using the pre-S1 interval. All preprocessing and quantification procedures were performed in accordance with standard event-related potential analysis protocols; refer to Fundamentals of Event-Related Potentials by Steven Luck.

#### 2.2.5 FFT analysis

Two minutes of EEG data were extracted from the P50 and CNV tasks. The data were converted to EDF format using Curry 7 and imported into EEGLAB v2024.0 (running on MATLAB R2022a). A bandpass filter of 0.5–45 Hz was applied, with a sampling rate of 1,000 Hz. Power spectral density (PSD) was computed using Fast Fourier Transform (FFT) with Hanning windows applied to 2-s epochs and 50% overlap. PSD was analyzed for five brain regions: bilateral frontal (FL/FR), parietal-occipital (OPL/OPR), temporal (TL/TR), and central (C). The frequency bands of interest included delta (1–4 Hz), theta (4–8 Hz), alpha (8–13 Hz), beta1 (13–20 Hz), and beta2 (20–30 Hz). For each band, power was calculated as the area under the PSD curve (e.g., integrating values from 1–4 Hz for the delta band) using the trapz function in MATLAB. This value reflects the cumulative oscillatory activity within each region and frequency band. Higher PSD values indicate stronger neural oscillatory activity in the corresponding frequency band within each brain region.

#### 2.2.6 Statistical analysis

Statistical analysis was conducted using SPSS Statistics 26 under the guidance of a professional statistician. Normality of data distribution was first assessed: P50 data did not conform to normal distribution, thus non-parametric tests were employed; CNV and FFT data exhibited normal distribution with homogeneity of variance, enabling independent samples t-tests. Statistical significance was set at *p* < 0.05. Additionally, two-tailed Pearson correlation analyzes examined relationships between P50 or CNV parameters and migraine clinical characteristics (including educational attainment, disease duration, attack duration, frequency, and pain severity).

## 3 Results

### 3.1 Evaluation of general clinical data

As shown in Table 1, in this study, no significant differences were observed between the patient and control groups in terms of age, gender, years of education, or BMI, indicating good comparability in these basic demographic characteristics. However, the patient group had significantly higher scores than the HC group on both the HAMA and HAMD scales.

### 3.2 ERP data analysis

#### 3.2.1 Analysis of P50 latency and amplitude in migraine patients

##### 3.2.1.1 S1-latency

**Figure 1:**
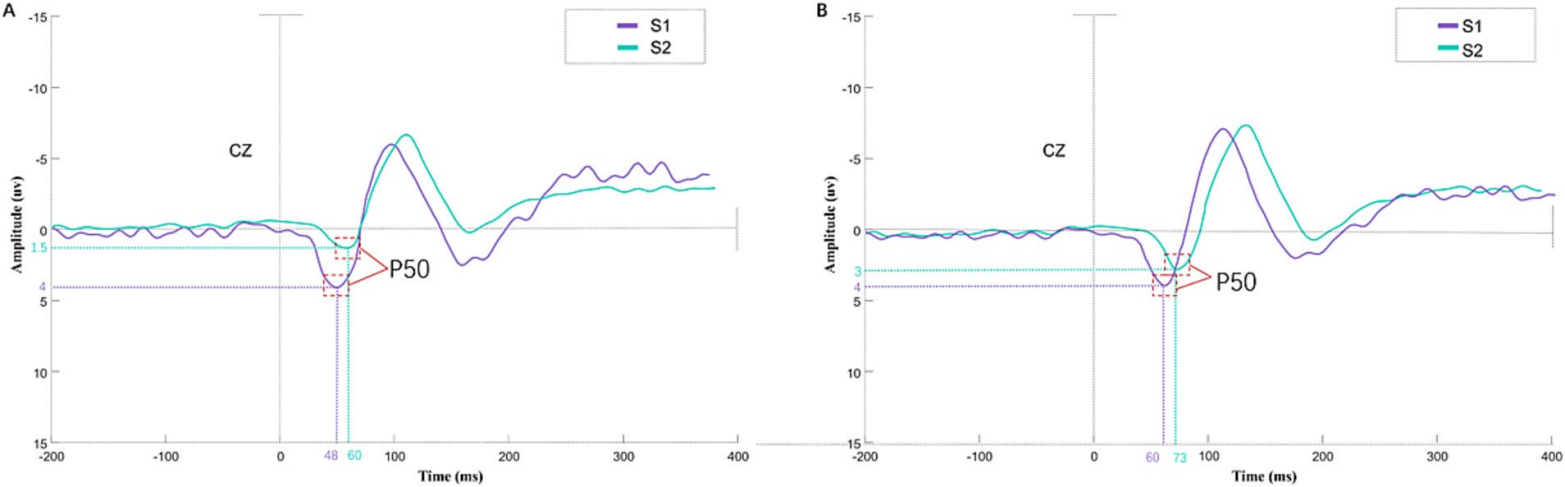
S1 and S2 evoked P50 ERP waveforms in healthy controls and migraine patients. **(A)** shows the healthy control group, while **(B)** depicts the migraine patient group. Using the Cz electrode as an example, this figure displays the P50 component ERP waveforms elicited by paired stimuli (S1, S2) under the paired-click paradigm. The purple waveform represents S1-evoked activity, and the cyan waveform indicates S2-evoked activity. The corresponding dashed lines mark the peak latency and amplitude positions of the P50 component. The superimposed S1 and S2 waveforms visually demonstrate the inhibitory effect of repeated stimulation: In the control group, S2 amplitude is significantly reduced compared to S1, indicating normal sensory gating function. In the migraine group, the inhibitory effect on S2 amplitude is weakened, reflecting impaired sensory gating function.

**Figure 2:**
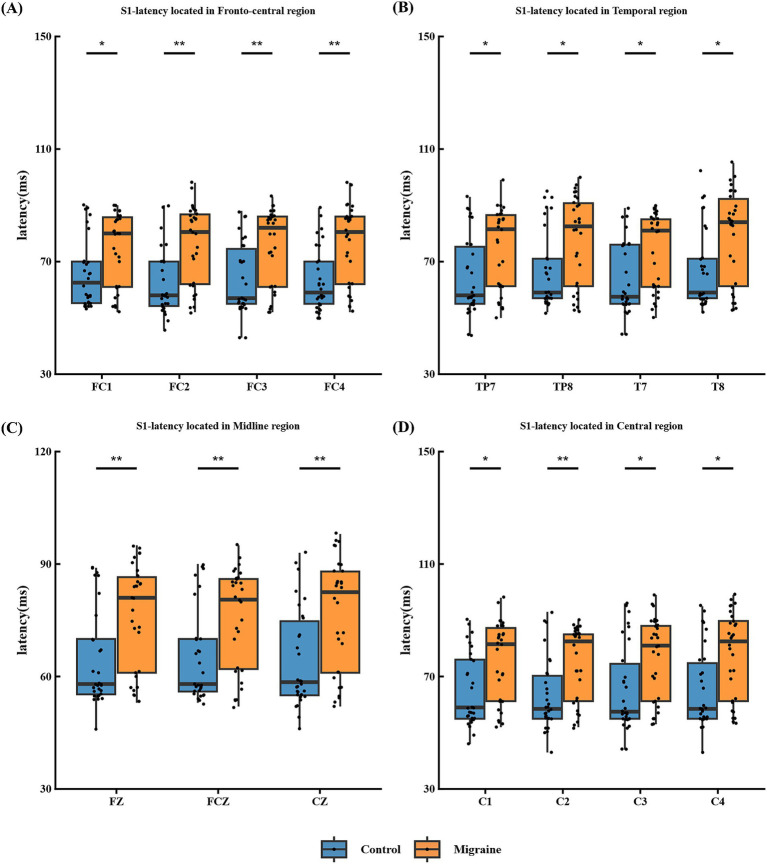
S1-latency in different regions of the P50 paradigm in migraine group and the control group. **(A)** S1-latency located in frontal central area FC1, FC2, FC3, and FC4; **(B)** S1-latency located in temporal area TP7, TP8, T7, and T8; **(C)** S1-latency located in midline area FZ, FCZ, and CZ; **(D)** S1-latency located in central area C1, C2, C3, and C4. There are significant differences in S1-latency between migraine group and control group in different areas (*p < 0.05; **p < 0.01; ***p < 0.001).

Figure 1 shows a schematic diagram of P50. Compared with the control group, patients exhibit prolonged P50 latency and a smaller decrease in S2 amplitude. The most positive peak between 30 and 90 milliseconds after stimulation is defined as S1, and the latency of S1 in the patient group is prolonged in the frontal central area FC1, FC2, FC3 and FC4 (Figure 2A), temporal area TP7, TP8, T7 and T8 (Figure 2B), mid-range area FZ, FCZ and CZ (Figure 2C) and central area C1, C2, C3 and C4 (Figure 2D).

##### 3.2.1.2 S2-latency

**Figure 3:**
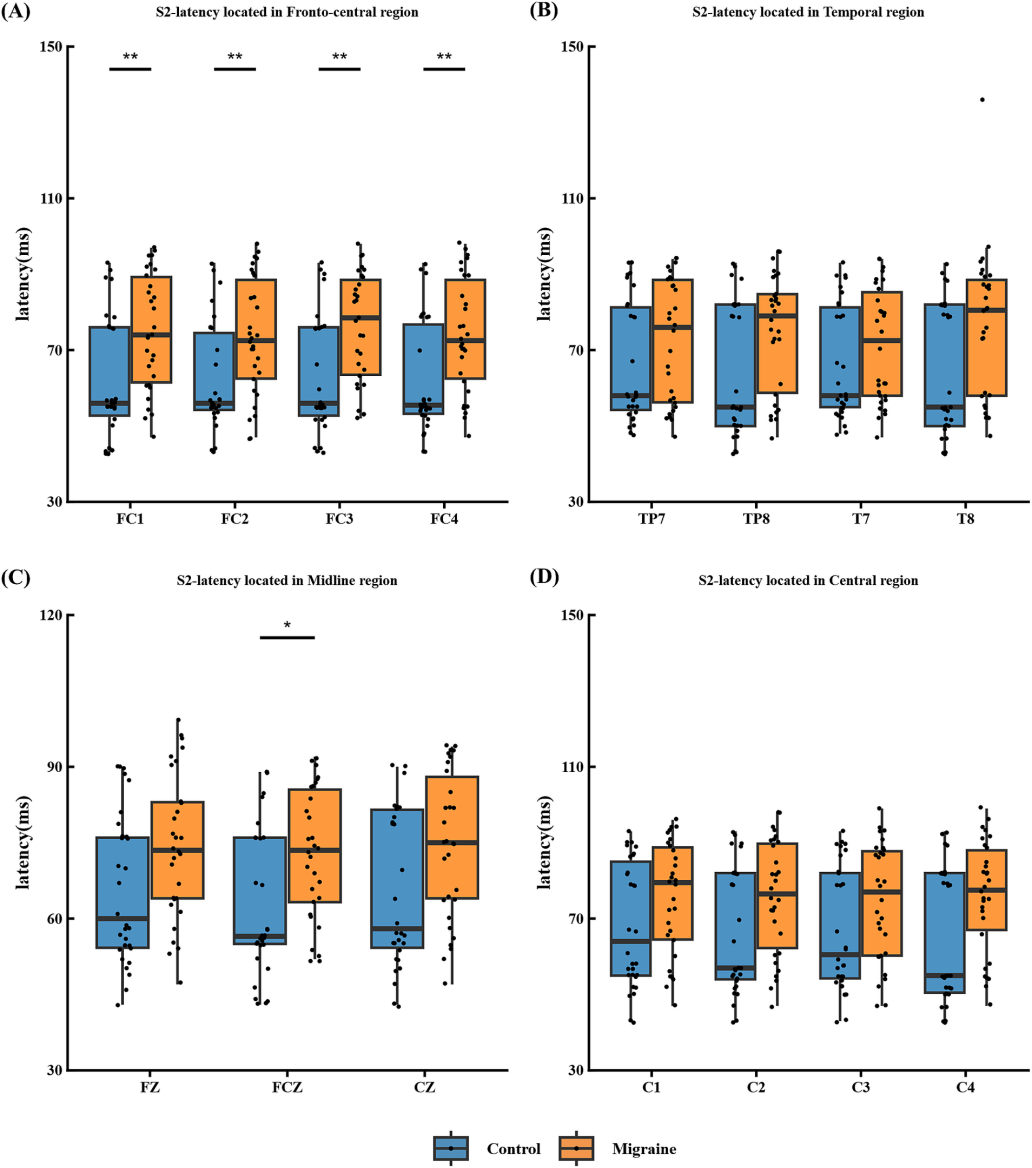
S2-latency in different regions of the P50 paradigm in migraine group and the control group. **(A)** S2-latency located in frontal central area FC1, FC2, FC3 and FC4; **(B)** S2-latency located in temporal area TP7, TP8, T7 and T8; **(C)** S2-latency located in midline area FZ, CZ and FCZ; **(D)** S2-latency located in central area C1, C2, C3 and C4. In the P50 test, there were significant differences in S2 latency between migraine group and control group in different areas (*p < 0.05; **p < 0.01; ***p < 0.001).

The P50 component of S2 is defined as the largest positive wave occurring within a 10-millisecond window following the P50 latency of S2 and the latency of S2 in the patient group is prolonged in the frontal central area FC1, FC2, FC3 and FC4 (Figure 3A), temporal area TP7, TP8, T7 and T8 (Figure 3B), Mid-range area FZ, FCZ and CZ (Figure 3C) and central area C1, C2, C3 and C4 (Figure 3D).

##### 3.2.1.3 Amplitude ratio: S2/S1

**Figure 4:**
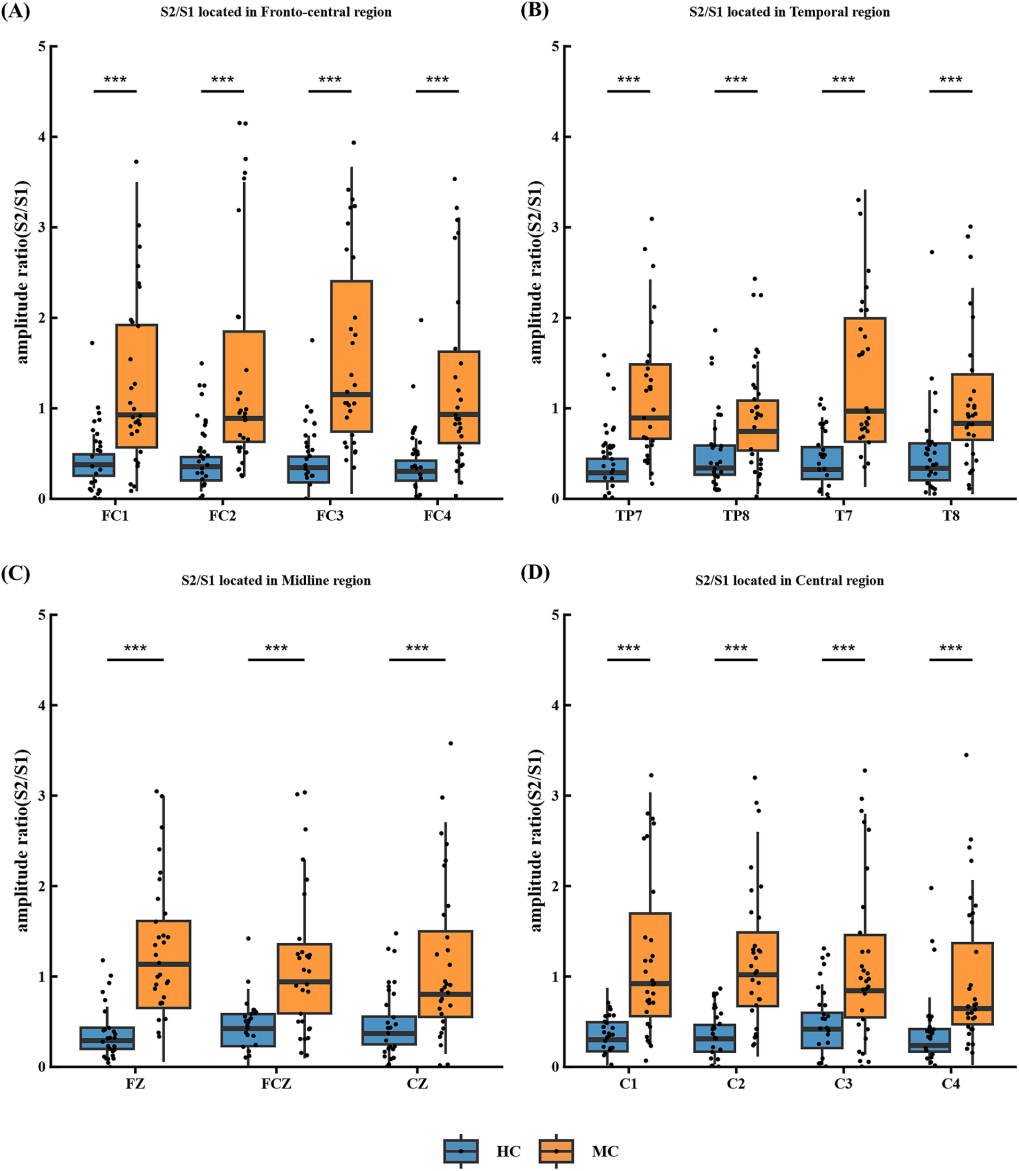
S2/S1 in different regions of the P50 paradigm in migraine group and the control group. **(A)** S2/S1 located in frontal central area FC1, FC2, FC3 and FC4; **(B)** S2/S1 located in temporal area TP7, TP8, T7 and T8; **(C)** S2/S1 located in midline area FZ, CZ and FCZ; **(D)** S2/S1 located in central area C1, C2, C3 and C4. In the P50 test, there were significant differences in S2/S1 between migraine group and control group in different areas (*p < 0.05; **p < 0.01; ***p < 0.001).

S2/S1 reflects cortical inhibitory capacity, with values below 0.5 in healthy individuals and above 0.5 in migraine patients. Compared to healthy controls, patients exhibited significantly increased S2/S1 ratios in the frontal central regions FC1, FC2, FC3, and FC4 (Figure 4A), temporal regions TP7, TP8, T7, and T8 (Figure 4B), Mid-range area FZ, FCZ and CZ (Figure 4C) and central area C1, C2, C3 and C4 (Figure 4C).

#### 3.2.2 CNV latency and amplitude analysis

##### 3.2.2.1 A-latency

**Figure 5:**
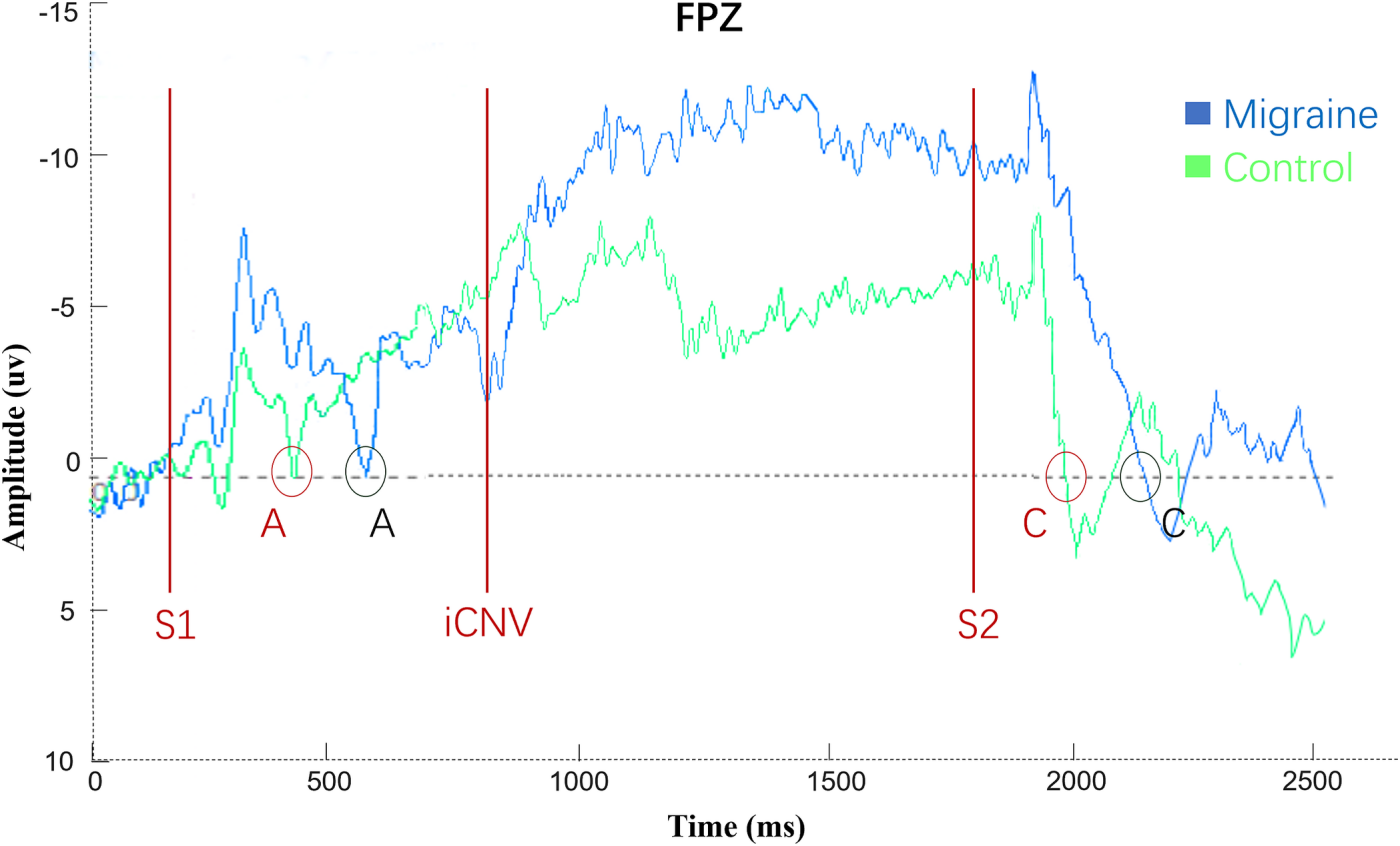
Difference of CNV latency, CNV amplitude, and CNV square between migraine group and control group under repeated auditory stimulation. oCNV, iCNV, and tCNV CNV square values were computed over the same time windows as their corresponding amplitude measures, PINV CNV square was defined as the integrated negative potential between S2 onset and point C.

**Figure 6:**
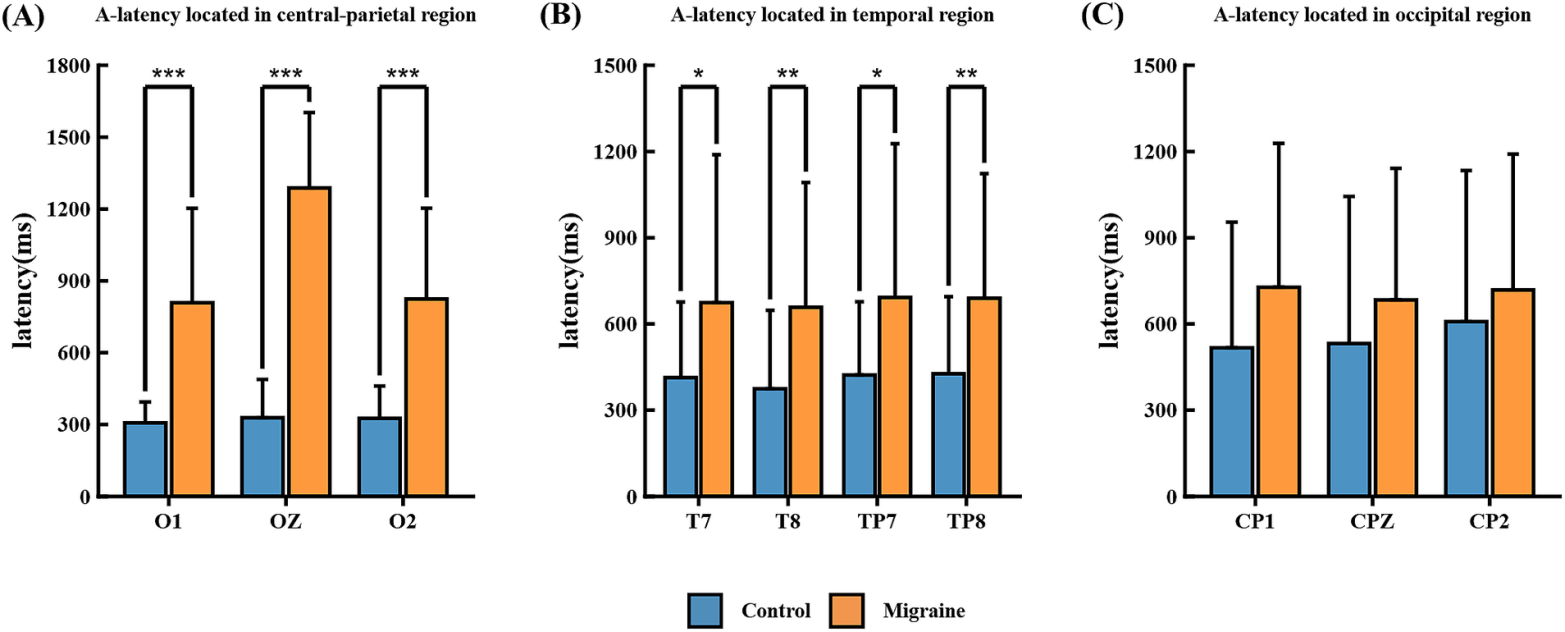
A-latency in different regions of the CNV paradigm in migraine group and the control group. **(A)** A-latency located in occipital O1, OZ, and O2; **(B)** A-latency located in temporal T7, T8, TP7, and TP8; **(C)** A-latency located in central-parietal CP1, CPZ, and CP2. A-latency between migraine and control groups located in different regions. A significant difference in A-latency was found between the migraine and control groups (*p < 0.05; **p < 0.01; ***p < 0.001).

Compared with the healthy control group, the A latency and C latency of patients are prolonged, and the area is enlarged. As shown in Figure 5. Point A is defined as the point where the post-S1 waveform crosses the baseline in a negative-going direction. The A-latency is measured as the time interval from S1 to Point A. Statistical analysis of the CNV revealed significantly prolonged A-latency in the patient group compared to the healthy controls across occipital O1, OZ, and O2 (Figure 6A), temporal T7, T8, TP7, and TP8 (Figure 6B), and central-parietal CP1, CPZ, and CP2 (Figure 6C).

##### 3.2.2.2 C-latency

**Figure 7:**
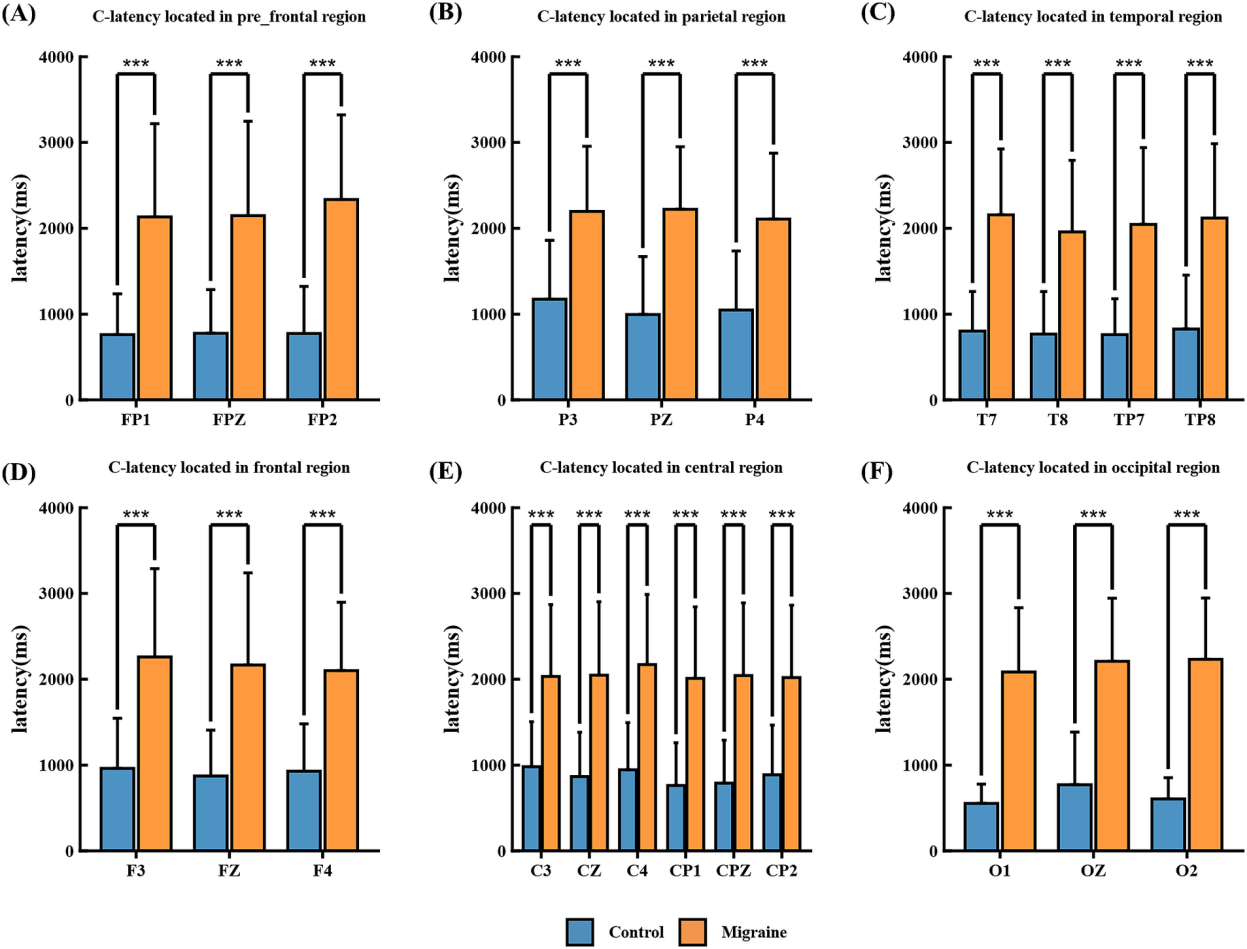
C-latency in different regions of the CNV paradigm in migraine group and the control group. **(A)** C-latency located in prefrontal FP1, FPZ, FP2; **(B)** C-latency located in parietal P3, PZ, and P4; **(C)** C-latency located in temporal T7, T8, TP7, and TP8; **(D)** C-latency located in frontal F3, FZ, and F4; **(E)** C-latency located in central C3, CZ, C4, CP1, CPZ, and CP2; **(F)** C-latency located in occipital O1, OZ, and O2. C-latency between migraine and control groups located in different regions. A significant difference in A-latency was found between the migraine and control groups (*p < 0.05; **p < 0.01; ***p < 0.001).

Point C was defined as the point where the post-S2 waveform crosses the baseline in a positive-going direction. The C-latency was measured as the time interval from S2 to Point C. Results revealed that the C-latency in the migraine group was significantly prolonged across multiple brain regions, including prefrontal regions FP1, FPZ, FP2 (Figure 7A), parietal regions P3, PZ, and P4 (Figure 7B), temporal regions T7, T8, TP7, and TP8 (Figure 7C), frontal regions F3, FZ, and F4 (Figure 7D), central regions C3, CZ, C4, CP1, CPZ, and CP2 (Figure 7E), and occipital regions O1, OZ, and O2 (Figure 7F).

##### 3.2.2.3 iCNV-latency

**Figure 8:**
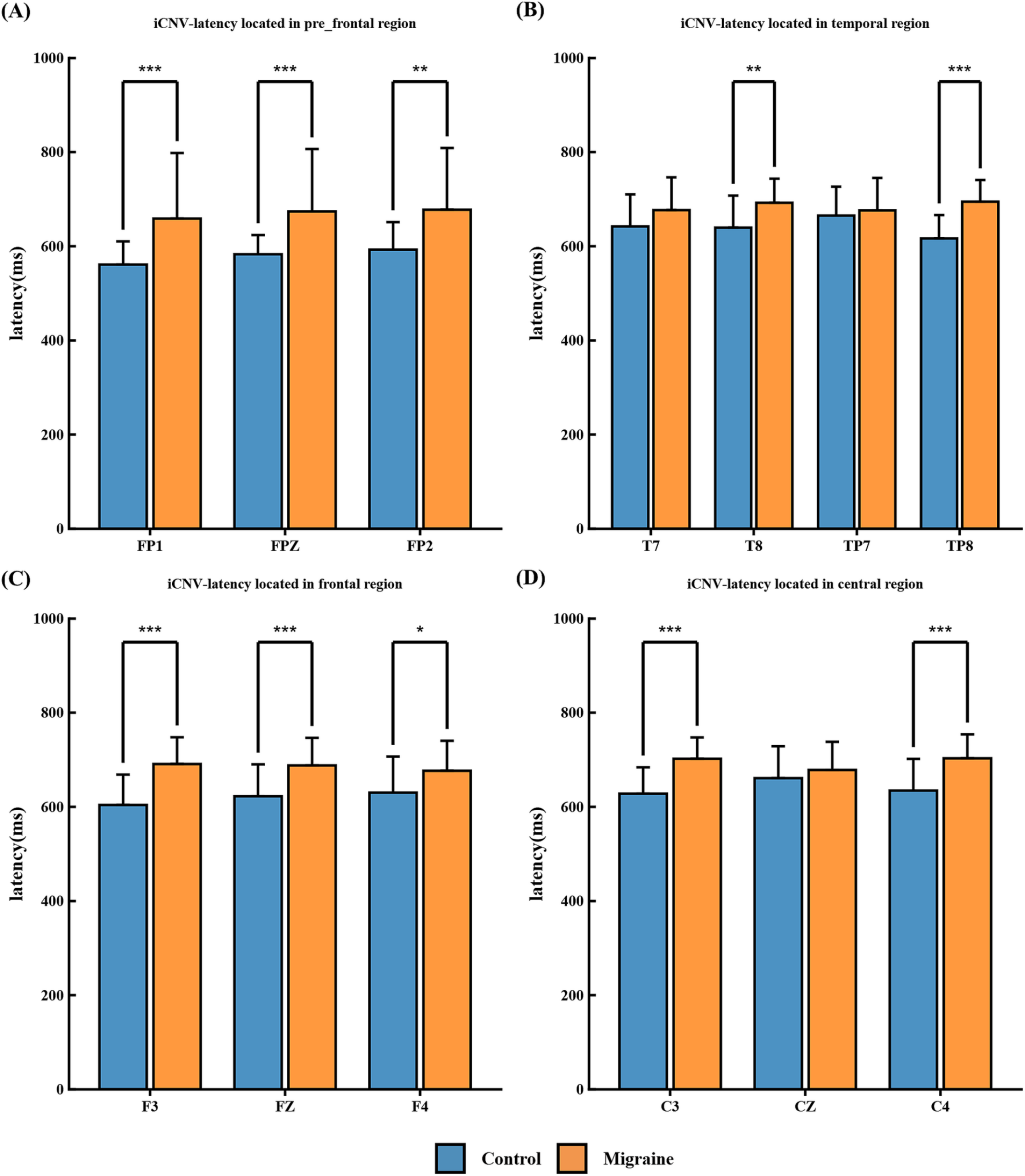
iCNV-latency in different regions of the CNV paradigm in migraine group and the control group. **(A)** iCNV-latency located in pre-frontal regions FP1, FPZ, and FP2; **(B)** iCNV-latency located in temporal regions T7, T8, TP7, and TP8; **(C)** iCNV-latency located in frontal regions F3, FZ, and F4, **(D)** iCNV-latency located in central regions C3, CZ, C4. iCNV-latency between migraine and control groups located in different regions. A significant difference in iCNV-latency was found between the migraine and control groups (*p < 0.05; **p < 0.01; ***p < 0.001).

The initial association negative latency (defined as the duration from S1 to the peak amplitude of iCNV) measured within the 500–1,000 ms interval following S1 stimulation is shown in the figure: The migraine group exhibited significant differences in the pre-frontal regions FP1, FPZ, and FP2 (Figure 8A), the temporal regions T7, T8, TP7, and TP8 (Figure 8B), frontal regions F3, FZ, and F4 (Figure 8C), and central regions C3, CZ, C4, (Figure 8D).

##### 3.2.2.4 iCNV-square

**Figure 9:**
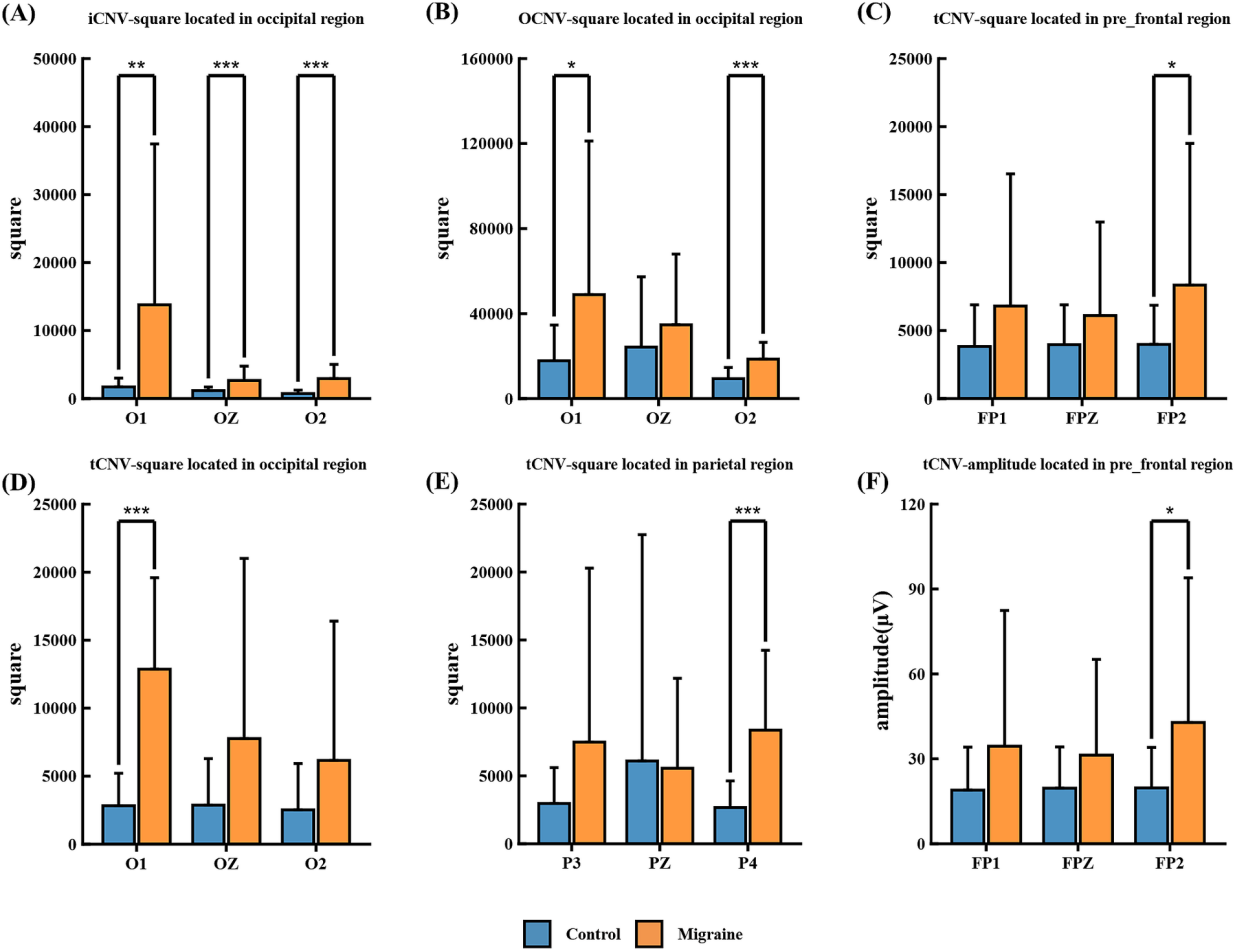
Different amplitudes and square components between the migraine and control groups. **(A)** iCNV-square located in occipital regions O1, OZ, and O2; **(B)** oCNV-square located in occipital regions O1, OZ, and O2; **(C)** tCNV-square located in pre-frontal regions FPI, FPZ, and FP2; **(D)** tCNV-square located in occipital regions O1, OZ, and O2; **(E)** tCNV-square located in parietal regions P3, PZ, and P4; **(F)** tCNV-ampitude located in pre-frontal regions FPI, FPZ, and FP2. Different amplitudes and square components between the migraine and control groups. A significant difference in CNV amplitude was found between the migraine and control groups (*p < 0.05; **p < 0.01; ***p < 0.001). A significant difference in CNV-square was found between the migraine and control groups (*p < 0.05; **p < 0.01; ***p < 0.001).

iCNV-square represents the negative change area from S1 to the iCNV point. The migraine group exhibited significantly greater iCNV-squared in occipital regions O1, O2 and OZ (Figure 9A) compared to the control group.

##### 3.2.2.5 oCNV-square

oCNV-square denotes the negative change area from S1 to S2. The oCNV-square in the migraine group was significantly greater than that in the control group in occipital regions O1, OZ, and O2 (Figure 9B).

##### 3.2.2.6 tCNV-square

tCNV-square represents the area of negative change occurring 200 ms prior to S2 onset. The migraine group exhibited significantly greater tCNV-square in pre-frontal regions FP1, FPZ, and FP2 (Figure 9C), occipital regions O1, OZ and O2 (Figure 9D), parietal regions P3, PZ and P4 (Figure 9E) compared to the control group.

##### 3.2.2.7 tCNV-amplitude

Terminal CNV (tCNV) represents the average amplitude detected 200 milliseconds prior to S2 onset. Significant differences in tCNV amplitude were observed between the migraine group and the control group at prefrontal regions FP1, FPZ, FP2 (Figure 9F).

### 3.3 FFT data

#### 3.3.1 Power spectrum analysis of P50 task in patient and control groups

**Figure 10:**
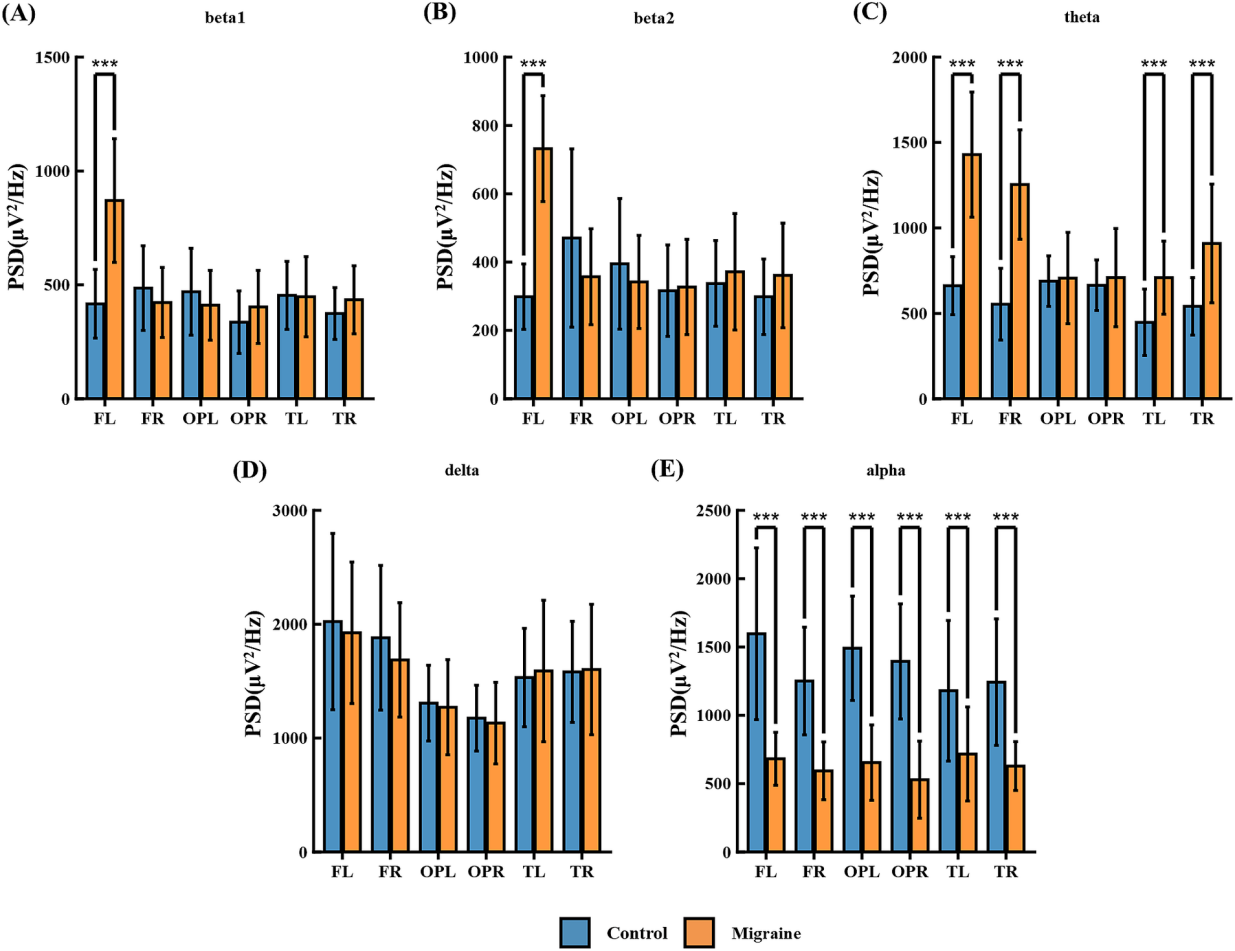
PSD comparison of each frequency band in two groups of P50 tasks. **(A)** beta1 located in the ares of FL, FR, OPL, OPR, TL, and TR; **(B)** beta2 located in the ares of FL, FR, OPL, OPR, TL, and TR; **(C)** theta located in the ares of FL, FR, OPL, OPR, TL, and TR; **(D)** delta located in the ares of FL, FR, OPL, OPR, TL, and TR; **(E)** alpha located in the ares of FL, FR, OPL, OPR, TL, and TR. FL is the left frontal area, FR is the right frontal area, OPL is the left occipital area, OPR is the right occipital area, TL is the left temporal area, TR is the right temporal area, and C is the central area (*p < 0.05; **p < 0.01; ***p < 0.001).

Power spectrum analysis of EEG recordings during the P50 task revealed significant differences between the migraine and control groups across multiple frequency bands (Figure 10). Specifically: Beta1 was significantly increased in the left frontal region FL (Figure 10A); Beta2 was significantly increased in the left frontal region FL (Figure 10B); Theta waves in the frontal regions FL and FR and temporal regions TL and TR (Figure 10C); Alpha was significantly increased in the frontal regions FL, FR, temporal regions TL, TR, and parietal-occipital regions OPL, OPR (Figure 10E). Detailed statistical analysis results are shown in Table 2.

**Table 2:** PSD comparison of each frequency band in two groups of P50 tasks.

ERP	Location	Control	Migraine	P
Delta	FL	2023.01 ± 773.49	1924.21 ± 620.69	0.590
	FR	1880.92 ± 635.24	1686.39 ± 502.22	0.190
	OPL	1307.10 ± 332.10	1271.09 ± 417.12	0.710
	OPR	1175.70 ± 288.92	1131.69 ± 358.29	0.600
	TL	1531.74 ± 432.19	1589.17 ± 621.21	0.680
	TR	1581.49 ± 443.33	1602.36 ± 572.42	0.880
Theta	FL	661.78 ± 169.51	1429.1 ± 366.23^***^	**< 0.001**
	FR	553.58 ± 209.98	1253.89 ± 319.68^***^	**< 0.001**
	OPL	688.70 ± 148.14	706.58 ± 267.31	0.750
	OPR	664.63 ± 148.02	709.17 ± 287.86	0.450
	TL	447.54 ± 193.99	708.46 ± 213.80^***^	**< 0.001**
	TR	540.76 ± 168.10	909.24 ± 347.50^***^	**< 0.001**
Alpha	FL	1596.77 ± 628.14^***^	682.39 ± 193.36	**< 0.001**
	FR	1251.31 ± 393.93^***^	594.13 ± 211.48	**< 0.001**
	OPL	1490.49 ± 381.90^***^	653.84 ± 275.51	**< 0.001**
	OPR	1394.46 ± 421.37^***^	529.35 ± 281.60	**< 0.001**
	TL	1179.78 ± 514.41^***^	717.14 ± 343.90	**< 0.001**
	TR	1242.76 ± 462.54^***^	628.94 ± 178.17	**< 0.001**
Beta1	FL	416.88 ± 150.80	869.96 ± 271.23^***^	**< 0.001**
	FR	485.92 ± 186.03	422.66 ± 153.84	0.160
	OPL	469.97 ± 190.95	410.58 ± 153.20	0.190
	OPR	336.14 ± 136.93	403.44 ± 160.65	0.090
	TL	454.03 ± 149.42	448.34 ± 176.00	0.890
	TR	374.32 ± 113.90	434.56 ± 149.06	0.080
Beta2	FL	298.75 ± 95.60	731.94 ± 154.73***	**< 0.001**
	FR	470.58 ± 260.78*	357.23 ± 140.25*	**0.040**
	OPL	394.93 ± 191.39	341.82 ± 136.34	0.220
	OPR	316.25 ± 133.85	327.13 ± 139.42	0.760
	TL	337.47 ± 125.37	371.54 ± 170.26	0.380
	TR	298.47 ± 110.71	361.03 ± 152.90	0.070

FL is the left frontal area, FR is the right frontal area, OPL is the left occipital area, OPR is the right occipital area, TL is the left temporal area, TR is the right temporal area, and C is the central area (*p < 0.05; **p < 0.01; ***p < 0.001). Bold values indicate statistically significant differences.

#### 3.3.2 Power spectrum analysis of the patient and control groups during the CNV task

**Figure 11:**
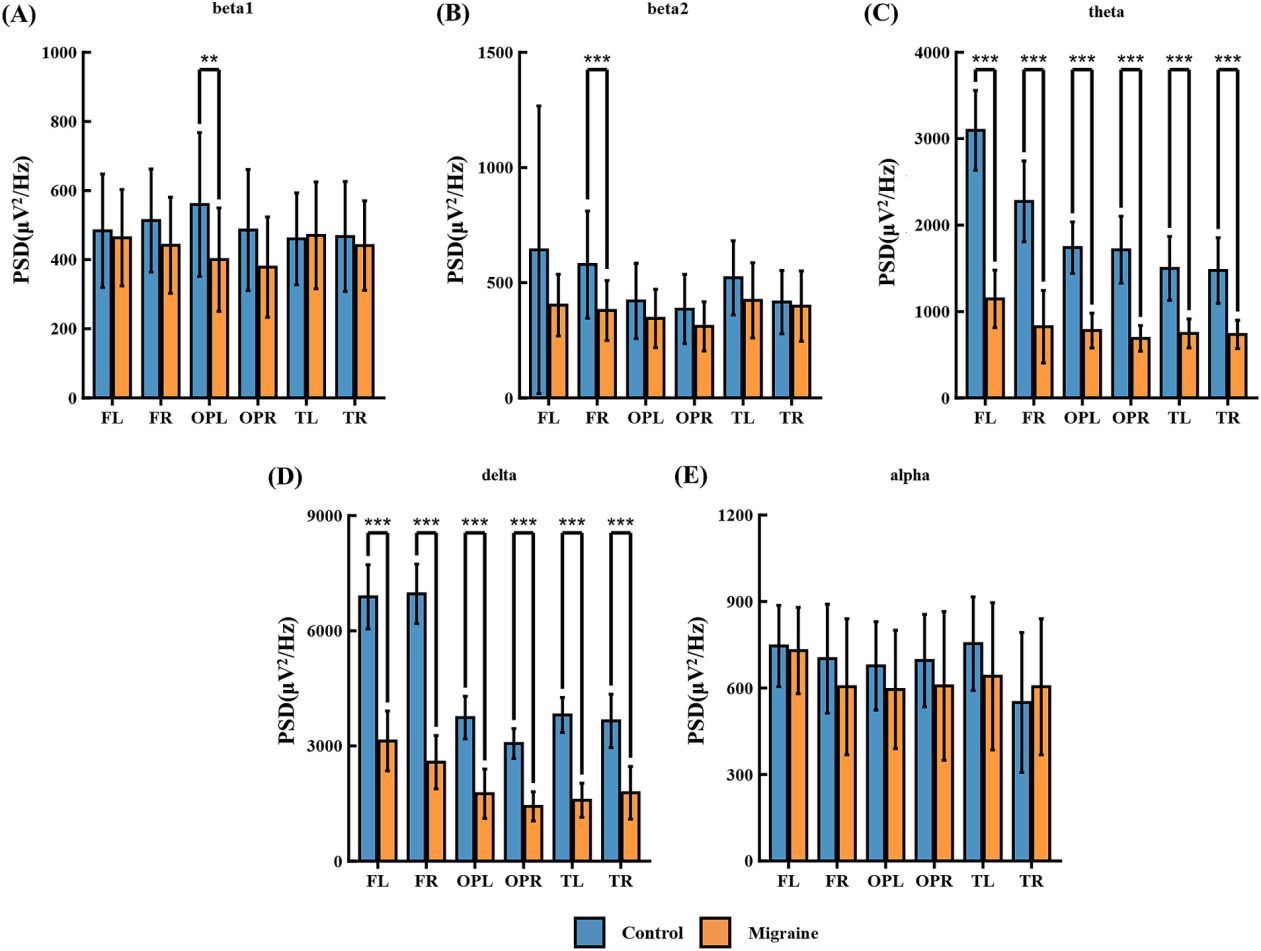
PSD comparison of each frequency band in two groups of CNV tasks. **(A)** beta1 located in the ares of FL, FR, OPL, OPR, TL, and TR; **(B)** beta2 located in the ares of FL, FR, OPL, OPR, TL, and TR; **(C)** theta located in the ares of FL, FR, OPL, OPR, TL, and TR; **(D)** delta located in the ares of FL, FR, OPL, OPR, TL, and TR; **(E)** alpha located in the ares of FL, FR, OPL, OPR, TL, and TR. FL is the left frontal area, FR is the right frontal area, OPL is the left occipital area, OPR is the right occipital area, TL is the left temporal area, TR is the right temporal area, and C is the central area (*p < 0.05; **p < 0.01; ***p < 0.001).

Power spectrum analysis of EEG signals during the CNV task revealed that, compared to the control group, the patient group exhibited beta1 was significantly increased in the left parietal-occipital regions OPL and OPR (Figure 11A); Beta2 significantly increased in the frontal regions FL, FR (Figure 11B); Theta waves were significantly increased in the frontal regions FL and FR, temporal regions TL and TR, and parietal-occipital regions OPL and OPR (Figure 10C); Delta waves in the frontal regions FL and FR, temporal regions TL and TR, and parietal-occipital regions OPL and OPR (Figure 10D); Alpha waves were significantly increased in the temporal region TL, and parietal-occipital regions OPL and OPR (Figure 10E). Detailed statistical analysis results are shown in Table 3.

**Table 3:** PSD comparison of each frequency band in two groups of CNV tasks.

ERP	Location	Control	Migraine	*P*
Delta	FL	6881.50 ± 835.58^***^	3129.90 ± 779.10	**< 0.001**
	FR	6959.40 ± 775.62^***^	2577.65 ± 696.01	**< 0.001**
	OPL	3738.08 ± 554.38^***^	1756.22 ± 642.17	**< 0.001**
	OPR	3062.11 ± 390.31^***^	1427.41 ± 381.77	**< 0.001**
	TL	3805.31 ± 456.57^***^	1588.15 ± 442.48	**< 0.001**
	TR	3654.64 ± 694.69^***^	1781.61 ± 686.22	**< 0.001**
Theta	FL	3095.97 ± 462.67^***^	1145.20 ± 333.13	**< 0.001**
	FR	2273.90 ± 467.90^***^	824.89 ± 420.06	**< 0.001**
	OPL	1738.99 ± 298.52^***^	779.56 ± 201.26	**< 0.001**
	OPR	1714.51 ± 387.15^***^	687.78 ± 148.31	**< 0.001**
	TL	1498.65 ± 370.59^***^	746.80 ± 167.44	**< 0.001**
	TR	1473.90 ± 380.40^***^	734.80 ± 164.37	**< 0.001**
Alpha	FL	745.61 ± 141.03	729.80 ± 149.52	0.680
	FR	701.61 ± 189.06	604.35 ± 236.02	0.080
	OPL	676.81 ± 153.24	595.04 ± 205.21	0.090
	OPR	695.13 ± 160.34	607.35 ± 257.69	0.120
	TL	753.71 ± 162.31^*^	640.73 ± 255.19	**0.050**
	TR	549.73 ± 242.85	604.35 ± 236.02	0.380
Beta1	FL	483.43 ± 164.43	463.33 ± 139.33	0.610
	FR	513.17 ± 149.09	441.49 ± 138.93	0.060
	OPL	559.19 ± 208.21^***^	400.04 ± 149.76	**< 0.001**
	OPR	485.45 ± 175.21^*^	378.38 ± 145.22	**0.010**
	TL	460.33 ± 133.08	470.18 ± 154.80	0.790
	TR	467.02 ± 159.10	440.93 ± 129.57	0.490
Beta2	FL	642.65 ± 624.06^*^	402.42 ± 133.75	**0.040**
	FR	578.76 ± 232.36^***^	379.44 ± 130.15	**< 0.001**
	OPL	420.69 ± 163.41^*^	344.92 ± 126.91	**0.050**
	OPR	385.40 ± 150.63^*^	310.42 ± 106.88	**0.030**
	TL	521.12 ± 161.09^*^	423.16 ± 163.16	**0.020**
	TR	415.70 ± 137.43	398.45 ± 152.68	0.650

FL is the left frontal area, FR is the right frontal area, OPL is the left occipital area, OPR is the right occipital area, TL is the left temporal area, TR is the right temporal area, and C is the central area (*p < 0.05; **p < 0.01; ***p < 0.001). Bold values indicate statistically significant differences.

## 4 Discussion

Migraine is a prevalent chronic neurological disorder with a high disability rate (35). Beyond headaches, nausea, and vomiting, its clinical presentation frequently includes cognitive impairment (36). Subjective cognitive decline is common among migraine sufferers; although cognitive symptoms are not core manifestations, many patients report intellectual deficits, particularly attention and memory impairments (37). Research indicates that migraine sufferers frequently experience cortical spreading depression (CSD), a potent wave of neuronal and glial depolarization that disrupts cortical function and induces persistent cerebrovascular dysfunction, thereby impairing cognitive abilities (38, 39). Previous studies have provided partial evidence supporting this direction: Gu et al. demonstrated that patients with cognitive impairment exhibited prolonged P50 latency and increased S2/S1 amplitude ratio during P50 tasks. Meanwhile, Ning et al. found that migraine patients showed amplified CNV amplitude, prolonged latency, and significantly increased area during CNV tasks (40, 41). Given the observed cognitive impairment, the present study selected two specific components from event-related potentials—P50 and CNV—to conduct an in-depth neurophysiological investigation. This analytical framework is grounded in the observation that migraine patients frequently exhibit imbalances in cortical excitability and inhibitory function. Their neural oscillatory characteristics may manifest as: reduced or asymmetrical Alpha band power, indicating insufficient inhibitory function and weakened attentional control. Thus, examining neural oscillatory activity in these brain regions and frequency bands may systematically reveal the neurophysiological mechanisms underlying migraine-related cognitive impairments. Furthermore, regarding the study subjects, the patient and control groups were rigorously matched for demographic characteristics. No statistically significant differences were observed between the two groups in key variables such as age, gender, body mass index (BMI), and years of education. This effectively controlled for potential confounding factors like educational attainment, ensuring comparability between the two samples.

This study found that migraine patients exhibit impaired sensory information processing across extensive brain regions, including frontal central (FC1, FC2, FC3, and FC4), central (C1, C2, C3, and C4), midline (FZ, CZ, and FCZ), and temporal (C5, C6, T7, and T8) areas. Although direct supporting literature remains scarce, these findings suggest migraine sufferers may have a generalized deficit in early sensory filtering and gating, resulting in reduced inhibition of repetitive stimuli (29, 34). P50 Sensory Gating Findings and Mechanisms: The auditory sensory gating mechanism primarily involves the thalamo-cortico-thalamic circuit regulated by the prefrontal cortex, particularly the inhibitory activity of GABAergic interneurons within it (42–44). Consequently, in paired-stimulus paradigms, a healthy neural system exhibits significant inhibition of the second stimulus (S2), resulting in a P50 amplitude substantially smaller than that of the first stimulus (S1). Neurophysiological investigations indicate an excitation-inhibition imbalance in the central nervous system of migraine sufferers, characterized by a generalized reduction in GABAergic inhibitory function (45, 46). This diminished inhibition directly impairs the brain’s ability to effectively ‘gate’ subsequent stimuli (S2), leading to an increased S2 amplitude and an elevated S2/S1 ratio. P50 perception abnormalities (manifested as elevated S2/S1 ratios) are closely associated with functional impairments in multiple core brain regions. Additionally, neuroimaging studies indicate that migraine patients exhibit structural abnormalities in brain regions involved in control and sensory integration, with these alterations closely associated with cognitive impairments. For instance, microstructural damage is observed in white matter tracts such as the corpus callosum, superior longitudinal fasciculus, and frontoparietal bundle (47, 48). These regions facilitate information transmission between cerebral hemispheres and between anterior and posterior brain areas, particularly affecting the prefrontal cortex’s ability to regulate sensory information. This impairs attentional and inhibitory functions. This widespread connectivity impairment—particularly reduced integrity in pathways between the prefrontal cortex and sensory cortices—diminishes signal transmission and integration efficiency within the sensory gating network. This constitutes the neural basis for abnormal P50 sensory gating (elevated S2/S1 ratio), providing electrophysiological evidence for understanding the dysfunction of early sensory filtering networks in migraine patients. Consequently, the increased P50 S2/S1 ratio provides compelling electrophysiological evidence for widespread functional abnormalities within the early sensory filtering network of the migraineur’s brain.

CNV, as a slow cortical event-related potential, exhibits distinct components corresponding to different cognitive processing stages (49). Consistent with previous studies, our findings reveal prolonged CNV latencies and increased wave amplitudes in migraine patients. Specifically, patients exhibited significantly longer A-latency, C-latency, and iCNV latency compared to healthy controls, with a whole-brain distribution. Among these, A-latency was most pronounced in occipital regions, iCNV latency primarily affected the frontal lobe, while C-latency showed a whole-brain distribution. Increased CNV amplitude was chiefly concentrated in the parietal and occipital regions (50). From an electrophysiological perspective, prolonged CNV latency may reflect delayed cognitive processing speed. For instance, Giesen S noted that the early component iCNV is a key indicator closely associated with attention and task preparation (4). The prolonged iCNV latency identified in this study suggests slowed processing during attention mobilization and stimulus preparation in migraine patients. Regarding amplitude, our results align with previous reports, demonstrating significantly increased (more negative) iCNV, oCNV, and tCNV amplitudes in migraine patients during the interictal period, particularly in parieto-occipital regions (P4, O1, and O2). CNV area metrics (e.g., oCNV-square, iCNV-square) were also generally enlarged, suggesting delayed habituation or weakened cortical inhibitory function. Differences in these area measures were primarily concentrated in occipital regions (51).

Methodologically, power spectral density analysis was employed to elucidate the frequency-domain characteristics of electroencephalographic activity, thereby deciphering the neural mechanisms underlying migraine-associated cognitive impairment (52). We selected the prefrontal cortex (F, responsible for executive function and attentional control), temporal lobe (T, involved in memory encoding and retrieval), and occipital-parietal region (OP, associated with visuospatial processing and attentional allocation) in the left and right hemispheres as regions of interest (53). Our analysis focused on the following frequency bands: Delta (*δ*, 0.5–4 Hz, associated with deep sleep and pathological states, typically reflecting impaired cognitive function), Theta (*θ*, 4–8 Hz, associated with drowsiness, memory encoding, emotional regulation, and attentional control), Alpha (*α*, 8–13 Hz, prominent during rest, reflecting relaxation and inhibitory control), and Beta (*β*, 13–30 Hz, linked to focus, cognitive processing, and motor planning, often further subdivided into β1 and β2) (54). During the NP50 task, significant differences in power spectral characteristics were observed between the migraine patient group and the healthy controls, reflecting abnormal neural functioning during complex information processing. The patient group exhibited heightened power in the theta and alpha bands across the prefrontal cortex, as well as increased beta 1 and beta 2 power specifically in the left frontal lobe. This pattern may indicate a functional imbalance during the recognition of redundant information, impairing the identification of repeated stimuli. The lateralised increase in beta power aligns with the established role of the left frontal lobe in dominating information recognition and cognitive processes (55). Within the temporal lobe, heightened theta and alpha power may indicate that migraine patients intensely concentrate on auditory stimuli due to an impaired ability to filter redundant information (56). As the superior temporal lobe houses the primary auditory cortex, the increased oscillatory activity in this region further supports this interpretation (57). During the CNV task, power spectral differences profoundly revealed neural dysfunction in processing anticipated information. In the prefrontal and temporal regions, which are key areas for cognitive control and anticipatory preparation, the patient group exhibited significantly heightened delta and theta band power. This reflects the greater cognitive effort required to sustain attention and task set, potentially indicating neural inefficiency. Meanwhile, heightened beta-band activity in the left frontal lobe may signify excessive activation during information recognition and internal processing, or an inability to effectively suppress extraneous neural activity, leading to functional imbalances in cognitive control (58). Moreover, extensive amplification of theta and alpha power within the temporal and parietal regions, which are centres for auditory processing and higher-order integration, further indicates the patients’ difficulty in effectively filtering redundant information. This necessitates the mobilization of greater neural resources for concentrating on auditory stimuli and sustaining anticipatory states, highlighting abnormalities in the sensory gating and attention maintenance networks (59).

Integration of ERP and FFT Findings: Combining ERP and FFT results provides a more comprehensive understanding of the neural basis underlying cognitive impairment in migraine patients. Abnormal P50 sensory gating (elevated S2/S1 ratio) reflects inhibitory deficits during early sensory filtering stages (60). The increased theta and alpha power observed in FFT analysis—particularly in the temporal lobes—further confirms that patients must mobilize excessive neural resources to process redundant stimuli during early sensory information processing. This hyperactivation may be directly linked to weakened GABAergic inhibition, leading to abnormal synchronization of neural oscillations (61).

Similarly, the slowing of cognitive processing speed and abnormal attention mobilization reflected by prolonged CNV latencies and increased amplitude are corroborated by the elevated delta/theta power in the prefrontal and temporal lobes observed in FFT analysis (62). This enhancement of low-frequency activity is typically associated with weakened cortical inhibitory function and reduced neural efficiency, suggesting that migraine patients require greater neural resources to maintain expected attention and execute control for the same level of cognitive tasks. The specific increase in left frontal beta power further reveals a pattern of hyperactivation in information recognition and internal processing (63).

## 5 Conclusion

This study systematically evaluated cortical function in migraine patients, revealing impaired sensory gating during the P50 task, delayed information processing, and heightened cortical excitability during the CNV task, accompanied by abnormal increases in theta and alpha band power across extensive brain regions. However, current research still faces several limitations, such as insufficient sample homogeneity, lack of integration with multimodal imaging data, and absence of longitudinal tracking designs, and lack of questionnaires assessing migraine-related quality of life and disability. To deepen mechanistic understanding and advance clinical translation, future studies will focus on expanding the scale of subtype-stratified samples, integrating brain network analysis with white matter structural exploration, and further investigating the efficacy and action pathways of neuromodulatory interventions, and incorporating validated quality-of-life and disability scales. This will provide more robust support for theoretical development and clinical practice in this field.

## Data Availability

The original contributions presented in the study are included in the article/Supplementary material, further inquiries can be directed to the corresponding author/s.
