# Correction: A study on cortical habituation based on event-related potential P50 and CNV

**DOI:** 10.3389/fneur.2026.1879326

**Published:** 2026-07-22

**Authors:** Jing Li, Yongxiang Zhang, Huanyu Li, Chang Liu, Zhiyuan Sun, Yunbo Fu, Qinghua He, Yudan Lv

**Affiliations:** 1 Neuroscience Center, Department of Neurology, The First Hospital of Jilin University, Changchun, China; 2 Headache Center, Department of Neurology, Beijing Tiantan Hospital, Capital Medical University, Beijing, China; 3 Changchun Institute of Optics, Fine Mechanics and Physics, Chinese Academy of Sciences, Changchun, China; 4 State Key Laboratory of CNS/ATM & MIIT Key Laboratory of Complex-field Intelligent Sensing, Beijing Institute of Technology, Beijing, China; 5 Guangdong Province Key Laboratory of Intelligent Detection in Complex Environment of Aerospace, Land and Sea, Beijing Institute of Technology, Zhuhai, China

**Keywords:** CNV, event-related potential, fast fourier transform, migraine, P50

In the published article there was an error in Table 1. Original incorrect values: Sample quantity: Migraine = 26, Control = 26. Male: Control = 34.61% Female: Control = 65.39% HADA: Migraine = 8.42 ± 1.85^**^ HAMD: Migraine = 7.93 ± 1.62^**^. The corrected Table 1 appears below.

**Table 1:** Evaluation of the general data between migraine and control groups.

General clinical data	Migraine	Control
Sample quantity	30	30
Age (years)	39.50 ± 16.51	38.88 ± 14.08
Male (%)	30.00	33.33
Female (%)	70.00	66.67
HAMA	4.86 ± 1.29	4.62 ± 1.40
HAMD	4.21 ± 1.25	4.81 ± 1.10
Pain intensity (VAS)	8.19 ± 0.78	–
Frequency of headache (per month)	9.19 ± 1.78	–
Education	13.02 ± 3.99	13.71 ± 3.40
Drug use during the study	–	–

HAMA, Hamilton Anxiety Rating Scale; HAMD, Hamilton Depression Rating Scale; VAS, Visual Analog Scale.

The original version of this article has been updated.

