# Supplementary material for: A study on cortical habituation based on event-related potential P50 and CNV
Source: Front Neurol. 2026 Apr 14;17:1787063. doi: 10.3389/fneur.2026.1787063 (PMC13121265; doi:10.3389/fneur.2026.1787063)
Supplement: Supplementary file 1 [file Table_1.docx]

Supplementary Material

# Supplementary Tables

**Table 1: S1 latency in different regions**

| Location | HC | CM | *P*-value |
| --- | --- | --- | --- |
| FZ | 64.60±12.87 | 76.23±14.07^**^ | 0.01 |
| CZ | 64.43±13.23 | 76.60±15.25^**^ | 0.01 |
| FCZ | 65.07±12.24 | 75.57±13.61^**^ | 0.01 |
| C1 | 65.07±13.07 | 75.70±14.25^*^ | 0.01 |
| C2 | 63.20±12.73 | 75.00±13.13^**^ | 0.01 |
| C3 | 65.33±15.51 | 76.53±14.59^*^ | 0.01 |
| C4 | 65.43±14.27 | 77.60±15.37^*^ | 0.01 |
| FC1 | 65.77±12.18 | 74.83±13.05^*^ | 0.02 |
| FC2 | 62.13±11.46 | 76.20±13.91^**^ | 0.01 |
| FC3 | 63.80±12.79 | 75.53±13.59^**^ | 0 |
| FC4 | 63.47±11.36 | 76.10±14.01^**^ | 0 |
| TP7 | 63.43±14.31 | 75.07±14.32^*^ | 0.04 |
| TP8 | 66.83±13.97 | 77.77±15.80^*^ | 0.04 |
| T7 | 63.43±12.73 | 73.43±13.78^*^ | 0.02 |
| T8 | 67.03±14.26 | 78.80±16.75^*^ | 0.04 |

**Table 2: S2 latency in different regions**

| Location | HC | CM | *P*-value |
| --- | --- | --- | --- |
| FZ | 65.83±14.78 | 74.23±14.34 | 0.05 |
| CZ | 65.73±15.43 | 74.47±14.59 | 0.05 |
| FCZ | 62.00±14.25 | 72.90±12.71^*^ | 0.01 |
| C1 | 68.23±16.46 | 75.50±14.92 | 0.1 |
| C2 | 66.33±16.56 | 75.67±15.08 | 0.1 |
| C3 | 67.10±16.42 | 74.57±15.58 | 0.1 |
| C4 | 65.83±17.63 | 75.87±14.75 | 0.1 |
| FC1 | 61.83±16.01 | 74.87±15.29^**^ | 0.01 |
| FC2 | 61.80±14.47 | 73.57±15.45^**^ | 0.01 |
| FC3 | 63.40±15.49 | 76.03±14.46^**^ | 0.01 |
| FC4 | 61.73±14.63 | 73.90±15.12^**^ | 0.01 |
| TP7 | 66.27±15.84 | 72.53±16.26 | 0.3 |
| TP8 | 64.97±17.49 | 74.70±15.28^*^ | 0.07 |
| T7 | 66.57±15.11 | 71.40±15.16 | 0.3 |
| T8 | 64.80±17.31 | 76.37±19.44^*^ | 0.06 |

**Table 3: S2/S1 in different regions**

| Location | HC | MC | P |
| --- | --- | --- | --- |
| C1 | 0.36±0.23 | 1.21±0.83^***^ | 0 |
| C2 | 0.37±0.29 | 1.24±0.78^***^ | 0 |
| C3 | 0.48±0.36 | 1.18±0.88^***^ | 0 |
| C4 | 0.39±0.41 | 0.96±0.75^***^ | 0 |
| FZ | 0.37±0.27 | 1.31±0.80^***^ | 0 |
| CZ | 0.46±0.33 | 1.13±0.85^***^ | 0 |
| FCZ | 0.44±0.31 | 1.14±0.75^***^ | 0 |
| FC1 | 0.44±0.30 | 1.21±0.88^***^ | 0 |
| FC2 | 0.45±0.37 | 1.39±1.17^***^ | 0 |
| FC3 | 0.38±0.29 | 1.51±1.05^***^ | 0 |
| FC4 | 0.39±0.33 | 1.25±0.92^***^ | 0 |
| TP7 | 0.41±0.36 | 1.12±0.73^***^ | 0 |
| TP8 | 0.52±0.43 | 0.93±0.59^***^ | 0 |
| T7 | 0.43±0.29 | 1.26±0.85^***^ | 0 |
| T8 | 0.50±0.48 | 1.09±0.71^***^ | 0 |
